# Alcohol use disorder and associated factors among individuals living with HIV in Hawassa City, Ethiopia: a facility based cross- sectional study

**DOI:** 10.1186/s13011-019-0212-7

**Published:** 2019-05-20

**Authors:** Bereket Duko, Alemayehu Toma, Yacob Abraham

**Affiliations:** 1 Faculty of Health Sciences, College of Medicine and Health Sciences, Hawassa University, P. O. Box 1560, Hawassa, Ethiopia; 2 Faculty of Medical Sciences, College of Medicine and Health Sciences, Hawassa University, P. O. Box 1560, Hawassa, Ethiopia

**Keywords:** Alcohol use disorder, Associated factors, HIV, AIDS, Ethiopia

## Abstract

**Background:**

Individuals living with HIV/AIDS with co-occurring harmful alcohol use may require specialized intervention or even multi-disciplinary team follow-up and management. This study was aimed to assess alcohol use disorder and associated factors among people living with HIV/AIDS in Hawassa city, Ethiopia, 2019.

**Methods:**

A facility based cross-sectional study was conducted among 195 people living with HIV/AIDS who had follow-up visit at Hawassa University Comprehensive Specialized hospital. A systematic sampling technique was used to recruit the study participants. Alcohol used disorders identification test (AUDIT) was used to measure alcohol consumption, drinking behaviors, and alcohol-related problems. The binary logistic regression model was used to see the association between alcohol use disorder (AUD) and the independent variables. The strength of association was measured by odds ratios with 95% confidence intervals. Statistical significance declared at *P* < 0.05.

**Results:**

The mean age of the study participants was 29.88 (±SD = 10.89) years. The magnitude of alcohol use disorder among people living with HIV/AIDS was 31.8%. Being male [AOR = 2.43, (95% CI: 1.76, 5.76)], having poor social support [AOR = 1.34, (95% CI: 1.12, 6.73)], being medication non-adherent [AOR = 1.78, (95% CI: 1.33, 6.79)], current khat chewing [AOR = 1.67, (95% CI: 1.16, 5.45)] and current cigarette smoking [AOR = 3.76, (95% CI: 2.16, 7.54)] had statistically significant association with alcohol use disorder.

**Conclusion:**

In the current study, magnitude of alcohol use disorder among people living with HIV was high and, calls for integrating services provided to HIV patients in HIV care and treatment clinic which enhances timely detection and management of AUD cases. This also alerts the stakeholders in HIV prevention and control programs to invest a greater efforts to retain patients in addiction treatment and rehabilitation centers. Lastly, appropriate screening and health education on consequences of alcohol use disorder is warranted.

## Background

Alcohol use disorder increases hospitalization and have negative effects on the life expectancy of patients infected with the human immunodeficiency virus (HIV) [1]**.** Alcohol can aggravate immunosuppression, impair cognitive function, often have detectable viral levels, and alcohol consumption may reduce patient adherence to ART, which can lead to treatment failure and leads to drug resistance to antiretroviral [2–5]**.** Moreover, hazardous alcohol use increases sexual risk behaviors for unprotected sex practice that leads to disseminating drug exposed virus to the public [2–6]**.**

Alcohol misuse is among in the group of problem in low-income countries including Ethiopia [7]**.** A study conducted on drug and alcohol abuse in Ethiopia revealed that the prevalence of hazardous alcohol use and alcohol dependence was 3 and 1.5% in general population respectively [8]**.** Different studies showed that higher prevalence of AUD among HIV patients; 32% in Ethiopia [9]**,** 38.4% in four African country [10]**,** 42% in Nepal [11]**,** 39.4% in Nigeria [12] and 74.3% in New York, USA [13]**.**

The most significant cause of ART interruption especially among men is harmful or hazardous alcohol use [5, 6]**.** The effectiveness of antiretroviral medication is highly dependent on patient adherence to highly active antiretroviral therapy (HAART) to significantly decrease HIV-related morbidity and mortality [3–5]**.** Being male sex, severe or moderate anxiety, being a college student, HIV related stigma and illicit drug use including tobacco are highly associated with alcohol use, and illicit drug use and CD4 count greater than or equal to 500 cells/microliter are highly associated with hazardous drug use [9–13]**.**

Health care providers are more often missing alcohol use problems in patients with less severe HIV infection and those without evidence of chronic diseases including liver disease [6]**.** Using specific instruments for assessing alcohol consumption enables the identification of less evident cases of alcohol abuse in HIV screening [14–19]**.** Patients with HIV/AIDS with hazardous or harmful alcohol use may require specialized intervention or even multi-disciplinary team follow-up and management [14–22]**.** This alerts the stakeholders in HIV prevention and control programs to invest greater efforts to retain patients in addiction treatment and rehabilitation centers [20, 21]**.**

Early detection of alcohol use disorder in HIV infected patients may strengthen the effectiveness of the medication which is significant for poor therapeutic adherence [20–22]**.** There is limited data concerning alcohol use disorder in HIV infected patients in Ethiopia for tailored prevention, diagnosis and treatment care in local context. Therefore, this cross-sectional study was aimed to assess the magnitude of alcohol use disorder and associated factors among patients with HIV/AIDS in Ethiopia.

## Methods

### Study design and setting

A facility based cross-sectional study was carried out to assess the epidemiology of alcohol use disorder and associated factors among patients living with HIV at Hawassa University Comprehensive Specialized Hospital, Hawassa, Ethiopia. This hospital is the only comprehensive specialized hospital found in Hawassa city, Ethiopia, which is situated 273 km from Addis Ababa, Ethiopia. It delivers both outpatient and inpatient services for more than 18 million population with more than 400 beds. In average, the hospital provides services for people living with HIV/AIDS at the ART clinic for more than 50 patients/day.

### Sample size determination and sampling procedure

Single population proportion formula was used to get the required sample size using prevalence of AUD among HIV patients in Ethiopia, 14.2% [23], with a 95% confidence interval and 5% of margin error. We used systematic sampling techniques to recruit the study participants through three intervals (K = 3). The first study participant among the 1st three participants selected through the lottery method. Among individuals who had a follow up visit at ART clinic, a total of 195 individuals with HIV who were with age of ≥18 years were selected for the study. Two study participants excluded from the study due to their hearing problems and critical stage of their illness.

### Data collection instruments and procedures

Four trained psychiatry nurses who had taken three days training on data collection had collected the data through an interviewer-administered questionnaire. Alcohol used disorders identification test (AUDIT) was used to measure alcohol consumption, drinking behaviors, and alcohol-related problems. It is a 10-items screening tool which was developed by WHO to identify alcohol use disorders for the last 12 months. The score of > eight or more in AUDIT was considered as alcohol use disorder. The scale has been validated in different ethnic groups and across genders with the sensitivity of 94.1% and specificity of 91.7%, is well-suited for use in primary care settings [24]**.** It was translated into Amharic which was the main languages spoken in the study area and back-translated to English by English language experts. It was tested in our pretest and highly reliable in our study (Cronbach’s alpha =0.94). Perceived HIV stigma scale was used to assess self-felt stigma by HIV patients. It is a Likert type of scale with four possible answers (strongly disagree = 1, disagree = 2, agree =3 and strongly agree = 4) to questions or statements on their opinions and feeling concerning how persons behave towards due to their HIV illness [25, 26]**.** ART medication adherence was looked at using the Morisky Medication Adherence Scale which is validated for the measure of medication non-adherence in a variety of patient populations and with over 110 versions and over 80 translations [27]**.** The Oslo 3-items social support scale was used to estimate the level of social support of the study participant’s percept. It is a scale with the sum score ranging from 3 to 14, 3–8 is” poor support”, 9–11 is” moderate support” and 12–14 is” strong support”. In this study, we consider good social support when the sum score is ≥9. It has been used in many population-based studies in Ethiopia [28]**.** Perceived HIV stigma, Morisky Medication Adherence Scale and Oslo social support scale were also highly reliable in this study with Cronbach’s alpha of 0.88, 0.92 and 0.94 respectively.

### Data processing and analyses

The checked and coded data was entered into Epi-Info version 7.0. Then transported to SPSS version 22 to analyze the data. Descriptive (frequency) statistics was done to get the distribution of socio-demographic, clinical and environmental factors and, a prevalence of AUD. The binary logistic regression model was used to see the association between AUD and the independent variables. Variables with a *P*-value of 0.20 and other variables of interest were entered into the multivariable logistic regression analysis. The strength of association was measured by odds ratios with 95% confidence intervals. Statistical significance was declared at *P* < 0.05.

## Results

### Socio-demographic characteristics of the study participants

A total of 195 individuals with HIV enrolled in the study with response rate of 98.9%. The mean age of the study participants was 29.88 (±SD = 10.89) years. Most of the study participants were in the age group of 35–44 years (40%), married (55.4) and living with their family (74.4%) (Table 1).

**Table 1:** Frequency for socio-demographic and socio-economic characteristics of the study participants, Hawassa, Ethiopia, 2019 (*n* = 195)

Characteristics	Category	Frequency	Percent (%)
Age	18–24 years	13	6.7
	25–34 years	55	28.2
	35–44 years	78	40.0
	≥ 45 years	49	25.1
Sex	Male	87	44.6
	Female	108	55.4
Religion	Orthodox	72	37.0
	Protestant	81	41.5
	Muslim	17	8.7
	Catholic	25	12.8
Marital status	Unmarried	39	20.0
	Married	108	55.4
	Divorced	32	16.4
	Widowed	16	8.2
Educational level	No formal education	6	3.1
	Grade 1–8	74	37.9
	Grade 9–12	69	35.4
	College and above	46	23.6
Occupation status	Civil servants	62	31.8
	Housewives	39	20.0
	Non-governmental	37	19.0
	Daily labor	32	16.4
	Student	25	12.8
Living condition	With family	145	74.4
	Alone	40	20.5
	With relatives	10	5.1
	Merchant	26	12.7
Average monthly income	< 1647 Ethiopian Birr	111	56.9
	1647–4537 Ethiopian Birr	53	27.2
	> 4537 Ethiopian Birr	31	15.9

### Clinical and psychosocial characteristics of the study participants

Among the respondents, 45.1 were with the illness duration of 5–10 years, 55% had good social support and 24.6% had TB/HIV coinfections (Table 2). Among the study participants who were classified as alcohol use disorder, 16.4% initiated drinking alcohol due to easily availability of alcohol, 13.8 and 11.8% started drinking alcohol due to peer pressure and lack of social support respectively (Table 3). Among those with AUD, 48.3% were preferred factory bottled beer, 35.4% were preferred locally brewed beer (‘tella’ and teji) and 19.4% were preferred arake.

**Table 2:** Clinical and psychosocial factors characteristics of the study participants, Hawassa, Ethiopia, 2019 (*n* = 195)

Variables	Category	Frequency	Percent %
Duration of the illness	< 5 years	49	25.1
	5–10 years	88	45.1
	≥ 10 years	58	29.7
CD4 count	< 500	114	58.5
	≥500	81	41.5
Social support	Good	108	55.4
	Poor	87	44.6
Body mass index (BMI) Kg/M^2^	18.5–24.99	134	68.7
	25–29.99	37	19.0
	> 30	24	12.3
Good medication adherence	Yes	170	87.2
	No	25	12.8
ART drug adherence	Poor	62	30.2
	Good	143	69.8
TB/HIV coinfection	Yes	48	24.6
	No	147	75.4
History of Hospital admission	Yes	61	31.3
	No	134	68.7
Past psychiatric history	Yes	16	8.2
	No	179	91.8
Family history of mental illness	Yes	12	6.2
	No	183	93.8
Past major surgery	Yes	26	13.3
	No	169	86.7
Current cigarette smoking	Yes	31	15.9
	No	164	84.1
Current chat (Khat) chewing	Yes	48	24.6
	No	147	75.4
Perceived stigma	Yes	58	29.7
	No	137	70.3

**Table 3:** Environmental and psychological factors associated with initiation of alcohol use disorders at Hawassa, Ethiopia, 2019 (*n* = 195)

Reasons	Frequency	Percent %
Easily availability of alcohol	32	16.4
Like the way alcohol makes feel happy	25	12.8
Peer pressure to drink	27	13.8
Parental modeling	16	8.2
Long standing life stressors	21	10.8
Lack of social support	23	11.8
Long lasting marital disharmony	13	6.7
Drinking to forget financial problems	15	7.7
To increase self confidence	14	7.2
To alleviate fear of socializing	10	5.1
Family history of alcohol use problem	18	9.2

### Prevalence of alcohol use disorder and associated factors

The prevalence alcohol use disorder among people living with HIV using AUDIT with cut-off point ≥8 was 31.8%. Finding from binary logistic regression analysis revealed that being male, having poor social support, current khat chewing, current cigarette smoking and poor medication adherence were statistically significant association with alcohol use disorder (Table 4).

**Table 4:** Factors associated with alcohol use disorder among people living with HIV/AIDS at Hawassa, Ethiopia, 2019 (*n* = 195)

Characteristics		AUD		Crude odds ratio (95% CI)	Adjusted odds ratio (95% CI)
		Yes	No		
Gender	Male	42	45	4.11, (2.34, 8.32)	2.43, (1.76, 5.76)**
	Female	20	88	1	1
Social support	Good	27	81	1	1
	Poor	35	52	2.01, (1.27, 7.25)	1.34, (1.12, 6.73)*
Good medication adherence	Yes	29	141	1	1
	No	9	16	2.73, (1.95, 8.87)	1.78, (1.33, 6.97)*
Current chat (khat) chewing	Yes	28	20	2.21, (1.35, 7.09)	1.67, (1.16, 5.45)**
	No	57	90	1	1
Current cigarette smoking	Yes	21	10	6.30, (2.35, 9.23)	3.76, (2.16, 7.54)**
	No	41	123	1	1
HIV perceived stigma	Yes	40	18	3.31, (1.01, 9.89)	0.96, (0.87, 5.12)
	No	55	82	1	1
Body mass index (BMI) Kg/M^2^	18.5–24.99	32	102	1	1
	25–29.99	15	22	2.17, (1.22, 6.38)	0.77, (0.72, 3.46)
	> 30	14	10	4.46, (2.32, 7.75)	0.93, (0.92, 5.57)

Reference category −1, * significant association (*p*-value < 0.05) **- significant association (*p*-value < 0.01)

## Discussion

In this study, the prevalence of alcohol use disorder among individuals with HIV was 31.8% (CI: 25.1–33.4). The prevalence of this study was in-line with the other studies in Ethiopia which was 32.6% [9]**,** 30.7% in Togo [10]**,** 25.7% in Nepal [29]**,** 28.6% in Brazil [30] and 27% in USA [31]**.** On the other hand, the prevalence of AUD is lower than the studies in China which is 42.8% [11] and higher than the studies from Ethiopia which is 14.2% [23] and 14 in Nigeria [32]**.** The difference in prevalence of alcohol use disorder could be due to the variation in the study setting, data collection instrument and the cutoff point of the instrument which was used to assess alcohol use disorder and the sample size in the study. In addition, the disparities in the magnitude might be due to variations in the underlying social, cultural and economic status among the study countries.

Males were 2.43 times more likely to have AUD when compared to females. This is in agreement with studies [9, 10, 23, 32]**.** Mostly, heavy drinking occurs among men when contrast to women and also biological differences among male and female could contribute to this difference [33]**.**

AUD was more commonly seen among those individuals with poor social support. This is in-agreement to the other studies [9–11]**.** This could be due to the fact that getting minimal social support from their neighbors or any person might cause dissatisfaction in their life. As a result, they might use alcohol to get rid of their stress and dissatisfaction.

Poor medication adherence had a significant association with alcohol use disorder. Individuals with HIV who had poor medication adherence were 1.78 times more likely to have alcohol use disorder. None of the studies proved the relationship between alcohol and medication adherence. Alcohol intoxicated individuals might forget to take the medications due to their memory impairments as result of deficiency in vitamin B1 (Thiamine) due to alcohol drinking [34]**.** On the other hand, they might use alcohol as a coping strategy for their psycho-social stressors associated with illness.

Chewing khat and smoking cigarette had a statistically significant association with AUD. This is in line with other studies in Ethiopia [9, 23] and four African countries [10]**,** USA [31] and China [11] respectively. This might be due to the fact that these substances are commonly interrelated with each other [34]**.** In addition to this, alcohol and cigarette (nicotine) could potentiate their rewarding effects and, cigarette smoking by itself bring down the intoxication and sedative effects of alcohol through stimulation [35]**.**

## Conclusion

In the current study, magnitude of alcohol use disorder among people living with HIV was high and calls for integrating services provided in HIV care and treatment clinic which enhances timely detection and management of AUD cases. Being male, having poor social support, current khat chewing, current cigarette smoking and poor medication adherence were statistically significant association with alcohol use disorder. This also alerts the stakeholders in HIV prevention and control programs to invest a greater efforts to retain patients in addiction treatment and rehabilitation centers. Lastly, appropriate screening and health education on consequences of alcohol use disorder is warranted.

### Limitation of the study

The study used standardized data collection instruments. However, some instruments are not validated to Ethiopian culture. This might over or under estimate the prevalence of AUD in this study. Because it was cross-sectional study design, it did not allow establishing a temporal relationship between AUD and other significantly associated factors.

## Abbreviations

AUD: Alcohol Use Disorder
AUDIT: Alcohol Used Disorders Identification Test
HAART: Highly Active Antiretroviral Therapy
